# Maggots as potential vector for pathogen transmission and consequences for infection control in waste management

**DOI:** 10.3205/dgkh000250

**Published:** 2015-05-06

**Authors:** Georg Daeschlein, Kevin Reese, Matthias Napp, Romy Spitzmueller, Peter Hinz, Michael Juenger, Axel Kramer

**Affiliations:** 1 Clinic of Dermatology, University Medicine, Ernst-Moritz-Arndt University Greifswald, Germany; 2 Department of Trauma and Orthopedic Surgery, Clinic of Surgery, University Medicine, Ernst-Moritz-Arndt University Greifswald, Germany; 3 Institute of Hygiene and Environmental Medicine, University Medicine, Ernst-Moritz-Arndt University Greifswald, Germany

**Keywords:** Lucilia sericata, maggot therapy, biosurgery, antisepsis, disinfection, larval disinfection, disposal of larvae

## Abstract

**Background and aims:** Debridement therapy with sterile bred larvae in non-healing wounds is a widely accepted safe and efficient treatment modality. However, during application in the contaminated wound bed microbial contamination with potential microbial pathogen spread after escape from the wound or after unreliable disposal procedure may happen, particularly in the case of not using bio-bags. The aims of this work were first to investigate the release of ingested bacteria into the environment by maggots and second to examine the common practice of freezing the maggots after use and/or disposal in trash-bags. Potential methods for hygienic safe disposal of used maggots should be deduced.

**Methods:** First, Maggots were contaminated with *S. aureus* by allowing them to crawl over an agar surface completely covered with bacterial growth over 24 h at 37°C. After external disinfection maggots were transferred onto sterile Columbia agar plates and shedding of *S. aureus* was visualized. Second, maggots were frozen at –20°C for 1, 2, 5, 10, 30, and 60 min. After exposure, the larvae were transferred onto Columbia blood agar with consecutive incubation at 37°C over 48 h. The larvae were analyzed visually for mobility and eating activities. The frozen bodies of dead larvae were examined for viable bacteria.

**Results:** We could demonstrate that maggots release formerly ingested pathogens (*S. aureus*). Freezing at –20°C for at least 60 min was able to kill all maggots, however the contaminant bacteria inside could survive.

**Conclusion:** Since freezing is apparently able to kill maggots but not to reliabely inactivate the ingested bacterial pathogens, we recommend the disposal of free-range larvae in screw cap vials after use to achieve full hygienic control.

## Zusammenfassung

**Zielsetzung:** Der Einsatz steriler handelsüblicher Larven zum Debridement chronischer Wunden ist ein akzeptiertes Therapieverfahren. Allerdings kann es bei Anwendung in der mikrobiell kontaminierten Wunde zur Weiterverbreitung von in der Wunde vorkommenden Erregern durch die Maden insbesondere nach unzuverlässiger Entsorgung kommen und wenn keine Biobags verwendet werden. Daher sollten sowohl die Freisetzung der von Maden ingestierten Bakterien in die Umgebung als auch die zur Inaktivierung der Maden übliche Praxis des Tiefgefrierens auf die ingestierten Bakterien vor der Entsorgung untersucht werden, um Schlussfolgerungen für eine sichere Entsorgung abzuleiten.

**Methode:** Maden wurden mit *S. aureus* kontaminiert, indem ihnen das Kriechen über eine komplett bewachsene Agarplatte nach Kultivierung für 24 h bei 37°C gewährt wurde. Nach äußerlicher Desinfektion wurden die Maden auf sterile Columbia Blutagarplatten überführt, um die Ausscheidung von *S. aureus* festzustellen.

Zur Überprüfung des Einflusses von –20 °C für 1, 2, 5, 10, 30 bzw. 60 min wurden die Maden auf Columbia Blutagarplatten überführt und 48 h bei 37°C kultiviert. Danach wurden die Mobilität und Fressaktivität der Maden visuell bewertet und der gefrorene Körper toter Maden wurde auf lebensfähige Bakterien untersucht.

**Ergebnisse:** Maden setzen zuvor ingestierte Bakterien (*S. aureus*) frei. Durch Gefrieren bei –20°C für mindestens 60 min werden die Maden abgetötet, während die ingestierten Bakterien überleben.

**Schlussfolgerung:** Da nur die Maden, nicht aber ingestierte Bakterien durch Einfrieren bei –20°C für 60 min abgetötet werden, ist die Entsorgung frei beweglicher Maden in Fläschchen mit Schraubverschluss zu empfehlen.

## Introduction

Bio-surgical debridement by calliphorid fly maggots was evolved in the 1920s [1]. With the improvement of surgical procedures and the implementation of novel antibiotic substances it got intermittently out of date since the 1940s [2], [3]. The method was re-introduced in 1988 for the treatment of chronic wounds [4], [5], [6], [7]. In face of the rising problems with multidrug-resistant pathogens due to antibiotic overuse the following decades it gained attention for its different modus operandi. Today it is an accredited method in the therapy of non-healing wounds.

Duration of maggot use (i.e. contact of larvae to the wound) is with bio-bags 3–4 days and with free-range larvae (50–400 maggots per treatment) 1–2 days [8], [9]. Following the treatment the bio-bags or the free-range larvae are disposed in trash-bags. The manufacturers do not give any recommendations on how to eliminate the maggots before disposal. Hence escaping larvae, which can be regularly observed using the free-range method, could reach other possibly not infected wounds of the treated patient or his/her direct environment or different hygiene sensitive areas in the hospital. Maggots are able to excrete viable pathogens such as methicillin-susceptible (MSSA) and methicillin-resistant *Staphylococcus aureus* (MRSA), which were ingested up to 48 h prior to excretion, as seen in a prior in vitro study, underscoring the potential role of used maggots as pathogen reservoir in hospitals [10]. Four cases of bloodstream infections with *Providencia stuartii* and one with *Candida albicans* were reported during maggot therapy, however, the blood isolates could be traced back to contaminated maggots of the species *Protophormia terraenovae*. The disinfecting procedure of the maggots was optimized and no case of sepsis occurred in 45 patients treated thereafter [11].

Accordingly, maggots used in debridement therapy must be regarded as potential reservoir and vector for pathogens and should be inactivated before disposal. Common practice in many hospitals is freezing the used larvae for an undetermined time-span at –20°C (as a disinfection procedure is not necessary for used wound dressings) to solve the obvious problem with these biological dressings of being able to move.

The aim of this work was to demonstrate the excretion of viable bacteria by maggots after ingestion and cuticula contamination and to evaluate the freezing method for inactivation of used maggots before disposal.

## Material and methods

### Maggots

The sterile maggots belonged to the species *Lucilia sericata*; *Diptera*: *Calliphoridae* (Biomonde GmbH, Barsbüttel, Germany). After visual examination and following incubation (48 h at 37°C on sterile Columbia agar plates with 5% sheep blood [Oxoid, Wesel, Germany]) they were used for the experiments in the third larval stage.

### Bacteria

Bacteria used in this study included a methicillin-susceptible strain of *Staphylococcus aureus* (MSSA, ATCC 6538, American Type Culture Collection, Manassas, Va., USA). All other used strains (*Escherichia coli*, *Enterococcus faecium*, *Pseudomonas aeruginosa*, *Proteus mirabilis*, *Klebsiella pneumoniae*, and methicillin-resistant strain of *Staphylococcus aureus* (MRSA)) were isolated from acute or chronic wounds of patients of our clinic during routine microbiology diagnostics or in the course of MRSA surveillance (admission screen for multidrug-resistant strains). Each strain was isolated from a different patient. Samples were processed following the national guidelines for microbiologic diagnostics. Identification and susceptibility testing were performed using the automated VITEKcompact system (Biomérieux, Nürtingen, Germany).

### Contamination of maggots with bacteria (MSSA) 

Ten maggots about 5 mm long and 3 mm in diameter were contaminated with MSSA by allowing them to crawl over an agar surface completely covered with bacterial growth over 24 h at 37°C. During exposition, all larvae (one per 90 mm agar plate) were monitored showing permanent crawling movements facing the complete agar surface and leaving deep spurs all over the agar surface caused by ingestion of bacterial colonies. Thereafter, the exterior of maggots was disinfected with 70% ethanol and exposure of 5 min. The larvae were rolled over ethanol-wetted paper towels with close contact of the complete larval cuticula (the larval disinfection was experienced in prior studies, where contact plates could show effective disinfection after 5 min exposure). The decontaminated maggots (control contact plates from each maggot did not show bacterial growth after 72 h, data not shown) were then transferred onto sterile Columbia agar plates for additional 24 h in order to visualize the shedding of bacteria by maggots. Additionally, 3 maggots prepared likewise were dissected (cut in tranches) and the tranches were put onto sterile Columbia agar plates.

### Maggot inactivation by freezing

To analyze deep freezing as inactivation tool of used maggots, maggots (10 larvae each) were frozen at –20°C for 1, 2, 5, 10, 30 and 60 min in sterile tubes (10 ml, Sarstedt, Germany). After exposure, the frozen larvae were transferred onto Columbia blood agar with consecutive incubation at 37°C over 48 h. The larvae were analyzed visually for mobility and eating activities. 

The same assay was performed with 5 larvae, which have been fed over 24 h on Columbia agar with colonies of *S. aureus* (MSSA), twelve fresh maggots contaminated with *E. coli*, *E. faecium*, *P. aeruginosa*, *K. pneumoniae*, *P. mirabilis*, and MRSA (2 maggots for each species) overnight by the same way, and five maggots taken from heavily microbial colonized chronic wounds (with *P. aeruginosa* and *P. mirabilis*) in which they had fulfilled their debridement activity. The frozen bodies of these larvae were examined for viable bacteria. This was executed by grinding the frozen bodies, streaking the obtained maggot powder onto Columbia blood agar and enumerating bacterial colonies after 24 h incubation (37°C) grown on the agar.

## Results

### Contamination of maggots with bacteria (MSSA)

**Figure 1:**
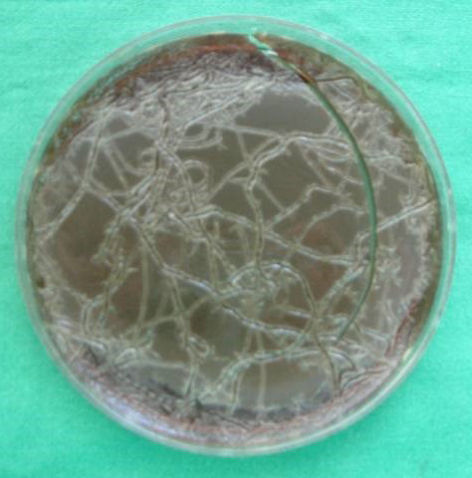
Spurs of MSSA on Columbia blood agar after 1 h of maggot creeping

All ten maggots exposed to MSSA on agar were shown to shed viable MSSA. The MSSA appeared as whitish spurs covering the agar surface with the pathogen after 24 h incubation (Figure 1 (Fig. 1)). Consecutively performed transversal cutting of three maggots revealed abundant staphylococcal growth (>10 CFU per tranche), showing that larvae of *L. sericata* are able to release viable MSSA, which were formerly ingested.

### Maggot inactivation by freezing

Freezing at –20°C for up to 30 min did not substantially affect the maggot’s vitality as shown by nearly unaffected viability of all maggots some minutes after thawing. Freezing for at least 60 min successfully killed all larvae (no mobility over 72 h after removal from freezer, data not shown).

The ingested bacteria of all tested species (MSSA, MRSA, *P. aeruginosa*, *P. mirabilis*, *E.coli*, *Klebsiella pneumoniae*, *E. faecium*) from the experimentally contaminated maggots and from the in real biosurgery contaminated as well, stayed active as anticipated, showing extensive growth on agar plates (data not shown).

## Discussion

The good results of maggot debridement therapy were commonly explained with controlled myiasis (i.e. debridement of necrotic tissue), stimulated tissue granulation [12], and secretion of antibacterial proteins [13]. Shortly after introduction of maggot debridement therapy by Baer in 1931 [1], the presence of an antibacterial substance in the body and secretions of *L. sericata* was demonstrated by Weil et al. [14] and since then the presence of several specific peptides with antibacterial activity, either in the body or the secretions of maggots were described [15], [16], [17]. The authors’ recent work showed that maggot secretion fulfills the definitions of an antiseptic and already small amount of larvae strongly affect bacteria [10]. Maggot excretions supposedly are free of bacteria as elimination of bacteria in the digestive tract to almost zero is described [18], [19]. In contrast, the authors’ recent work showed that ingested strains of MSSA and MRSA were excreted to the environment and even remained vital within the pupa [10]. However, other studies demonstrated that maggots of *L. sericata* are not able to eliminate equally well different Gram+ and Gram– bacteria [15], [16], [17], [20].

Despite their antibacterial activities, maggots themselves remain contaminated with bacteria (most probably around their mouthparts) and they are able to shed them to the environment (most probably together with their saliva). Accordingly, under certain circumstances and for given bacteria (i.e. MRSA and extended-spectrum beta-lactamase Gram-negative bacteria), maggots might serve as vectors of germ transmission, especially when they escape from the wound or are actively removed from wounds at the end of the bio-debridement therapy, without being properly disposed as clinical waste. The data at hand show that larvae ingest and release MSSA, even several days after stopping ingestion. Other bacteria may follow this way as it was shown for *P. mirabilis* and *P. aeruginosa* cultured from larvae, which were removed from patient’s wound, and for *K. pneumoniae*, *E. coli*, *E. faecium*, *P. aeruginosa*, and MRSA after artificial contamination. Accordingly, maggots separated from contaminated wounds are able to transfer wound pathogens to their environment by direct contact (body contamination) and indirectly by shedding viable pathogens. On the other hand, recontamination or infection of already cleansed wounds or wounds under debridement via contaminated maggots is possible. This risk should be focused by adequate measures: one could be antiseptic wound treatment in parallel to the maggot therapy, which until now not has been described and would be desirable in particular for expected cumulative beneficial effects. Another measure could be establishment of reliable de-waste procedures.

As for delineation of potential methods for safe inactivation of used maggots before disposal together with potentially viable ingested pathogens only freezing over at least 60 min at –20°C caused the death of all maggots, however the *in vitro* or on patient’s wound ingested bacteria were still viable. Therefore, the authors recommend disposal of free range maggots in closed vials (screw cap) and regard them as biological waste. Bio-bags can be discarded in normal trash-bags if the bio-bag is completely intact. A possible alternative may be inactivation of colonizing pathogens in parallel with maggot activity in the wounds when it can be demonstrated that antisepsis is not harming maggot activity. This is currently under investigation. 

## Conclusions

1. Used larvae should be regarded as biological waste and potentially will carry and spread multiple and multi-drug resistant pathogens.
2. Maggots can easily survive freezing for up to 30 minutes at –20°C. They can be killed when exposed over 60 min but preserve vital pathogens inside their body. The disposal of free-range larvae into screw capped vials easily achieves full hygienic control.
3. Bio-bags should get visually examined for intactness before disposal in normal trash-bags.

## Notes

### Authorship

G. Daeschlein and K. Reese contributed equally.

### Competing interests

All authors and co-authors deny any potential conflict of interest (e.g. employment, consulting fees, research contracts, stock ownership, patent licenses, honoraria, advisory affiliations, etc.).

## References

[1] Baer WS (1931). The treatment of chronic osteomyelitis with the maggot (larva of the blow fly). J Bone Joint Surg Am.

[2] Livingston SK, Prince LH (1932). The treatment of chronic osteomyelitis with special reference to the use of the maggot active principle. JAMA.

[3] McKeever DC (1933). Maggots in treatment of osteomyelitis. J Bone Joint Surg.

[4] Sherman RA, Pechter EA (1988). Maggot therapy: a review of the therapeutic applications of fly larvae in human medicine, especially for treating osteomyelitis. Med Vet Entomol.

[5] Mumcuoglu KY, Ingber A, Gilead L, Stessman J, Friedmann R, Schulman H, Bichucher H, Ioffe-Uspensky I, Miller J, Galun R, Raz I (1999). Maggot therapy for the treatment of intractable wounds. Int J Dermatol.

[6] Sherman RA (2002). Maggot versus conservative debridement therapy for the treatment of pressure ulcers. Wound Repair Regen.

[7] Jones M, Thomas S (1997). Wound cleansing – a therapy revisited. J Tissue Viability.

[8] Dumville JC, Worthy G, Bland JM, Cullum N, Dowson C, Iglesias C, Mitchell JL, Nelson EA, Soares MO, Torgerson DJ, VenUS II team (2009). Larval therapy for leg ulcers (VenUS II): randomised controlled trial. BMJ.

[9] Blake FA, Abromeit N, Bubenheim M, Li L, Schmelzle R (2007). The biosurgical wound debridement: experimental investigation of efficiency and practicability. Wound Repair Regen.

[10] Daeschlein G, Mumcuoglu KY, Assadian O, Hoffmeister B, Kramer A (2007). In vitro antibacterial activity of Lucilia sericata maggot secretions. Skin Pharmacol Physiol.

[11] Nuesch R, Rahm G, Rudin W, Steffen I, Frei R, Rufli T, Zimmerli W (2002). Clustering of bloodstream infections during maggot debridement therapy using contaminated larvae of Protophormia terraenovae. Infection.

[12] Prete PE (1997). Growth effects of Phaenicia sericata larval extracts on fibroblasts: mechanism for wound healing by maggot therapy. Life Sci.

[13] Fleischmann W, Grassberger M, Sherman R (2004). Maggot Therapy: A Handbook of Maggot-Assisted Wound Healing.

[14] Weil GC, Simon RJ, Sweadner WR (1933). A biological, bacteriological and clinical study of larval or maggot therapy in the treatment of acute and chronic pyogenic infections. Am J Surg.

[15] Huberman L, Gollop N, Mumcuoglu KY, Block C, Galun R (2007). Antibacterial properties of whole body extracts and haemolymph of Lucilia sericata maggots. J Wound Care.

[16] Bexfield A, Nigam Y, Thomas S, Ratcliffe NA (2004). Detection and partial characterisation of two antibacterial factors from the excretions/secretions of the medicinal maggot Lucilia sericata and their activity against methicillin-resistant Staphylococcus aureus (MRSA). Microbes Infect.

[17] Kerridge A, Lappin-Scott H, Stevens JR (2005). Antibacterial properties of larval secretions of the blowfly, Lucilia sericata. Med Vet Entomol.

[18] Robinson W, Norwood VH (1934). Destruction of pyogenic bacteria in the alimentary tract of surgical maggot implanted in infected wounds. J Lab Clin Med.

[19] Mumcuoglu KY (2001). Destruction of Bacteria in the Digestive Tract of the Maggot of Lucilia sericata (Diptera: Calliphoridae). J Med Entomol.

[20] Kruglikova AA, Chernysh SI (2011). Antimicrobial compounds from the excretions of surgical maggots, Lucilia sericata (Meigen) (Diptera, Calliphoridae). Entomologicheskoe Obozrenie.

